# GlycoRNA: A new player in cellular communication

**DOI:** 10.32604/or.2025.060616

**Published:** 2025-04-18

**Authors:** HYUNG SEOK KIM

**Affiliations:** Department of Biochemistry, Kosin University College of Medicine, Seo-gu, Busan, 49267, Republic of Korea

**Keywords:** GlycoRNA, Cancer biology, Glycosylation, Immune evasion, Therapeutic targets, RNA modifications, Lectin interactions

## Abstract

The discovery of glycosylated RNA molecules, known as glycoRNAs, introduces a novel dimension to cellular biology. This commentary explores the transformative findings surrounding glycoRNAs, emphasizing their unique roles in cancer progression and the therapeutic opportunities they present. GlycoRNAs, through interactions with lectins and immune receptors, may contribute to tumor immune evasion. Moreover, the therapeutic potential of this emerging knowledge includes interventions targeting glycoRNA synthesis and modulation of associated signaling pathways. By highlighting these critical insights, this commentary aims to encourage the development of innovative strategies that could improve cancer prognosis and treatment.

## Introduction

Molecular biology has been profoundly impacted by the discovery of glycosylated RNAs (glycoRNAs), which challenges the traditional view that glycosylation is exclusive to proteins and lipids. GlycoRNAs are characterized by the attachment of complex carbohydrates, including sialylated glycans, to RNA molecules, representing a previously unrecognized modification. Notably, glycoRNAs have been identified on cell surfaces, suggesting their involvement in cell-to-cell communication and immune recognition [1]. Their presence introduces a new layer of complexity to molecular interactions within cells and the extracellular environment.

Glycosylation patterns, including those of proteins and lipids, play critical roles in tumor progression and immune system interactions [2]. The glycosylation of RNA adds to this complexity, potentially influencing cancer cell behavior and immune responses within the tumor microenvironment [3]. The implications of glycoRNAs in cancer are significant, as they may contribute to mechanisms of immune evasion, metastasis, and therapy resistance. Although the biology of glycoRNAs is not yet fully understood, they are expected to emerge as a major research focus in the coming decades. This commentary explores these implications, discussing how glycoRNAs could serve as novel therapeutic targets and their potential impact on future cancer treatments.

## Discovery and Biology of GlycoRNA

The discovery of glycoRNAs was first reported by Flynn et al., who observed that small non-coding RNAs, including Y RNAs, small nuclear RNAs (snRNAs), ribosomal RNAs (rRNAs), small nucleolar RNAs (snoRNAs), and transfer RNAs (tRNAs), can be modified with N-linked glycans. These glycans contain sialylated structures commonly formed during protein glycosylation [1]. Mohorko et al. proposed that glycoRNA biosynthesis may involve glycosyltransferases traditionally associated with the modification of proteins and lipids. For instance, N-acetylgalactosaminyltransferases (GALNTs) may initiate O-glycan addition to RNA, while sialyltransferases could elongate these glycan chains with sialic acids. Additionally, oligosaccharyltransferases are hypothesized to transfer pre-assembled glycan blocks onto specific RNA modifications [4]. Although these enzymes are well characterized in the context of proteins and lipids, their precise substrate specificity, recognition motifs, and catalytic mechanisms when acting on RNA remain poorly understood, presenting a significant conceptual challenge. The exact mechanism of RNA glycosylation thus remains unclear.

It has been hypothesized that glycoRNA biosynthesis might occur through processes similar to the N-linked glycosylation of proteins, involving the endoplasmic reticulum (ER) and Golgi apparatus. However, this presents a paradox, as RNA molecules are not typically localized within these organelles. Several hypotheses have been proposed to address this issue. For example, certain RNA-binding proteins (RBPs) may chaperone RNAs into or near the ER/Golgi compartments, facilitating access to enzymatic glycosylation machinery. Alternatively, atypical trafficking routes may allow RNA or RNA-containing complexes to transiently interact with ER/Golgi-associated glycosylation enzymes. Recent reviews have highlighted unconventional vesicular transport, RNA-RBP complexes, and even localized translation of RBPs as potential mechanisms guiding RNA to glycosylation sites [5–7]. Further research is required to delineate the biochemical and cellular pathways enabling glycoRNA formation, as understanding these mechanisms is crucial for identifying potential therapeutic targets and modulators of glycoRNA synthesis.

**Figure 1:**
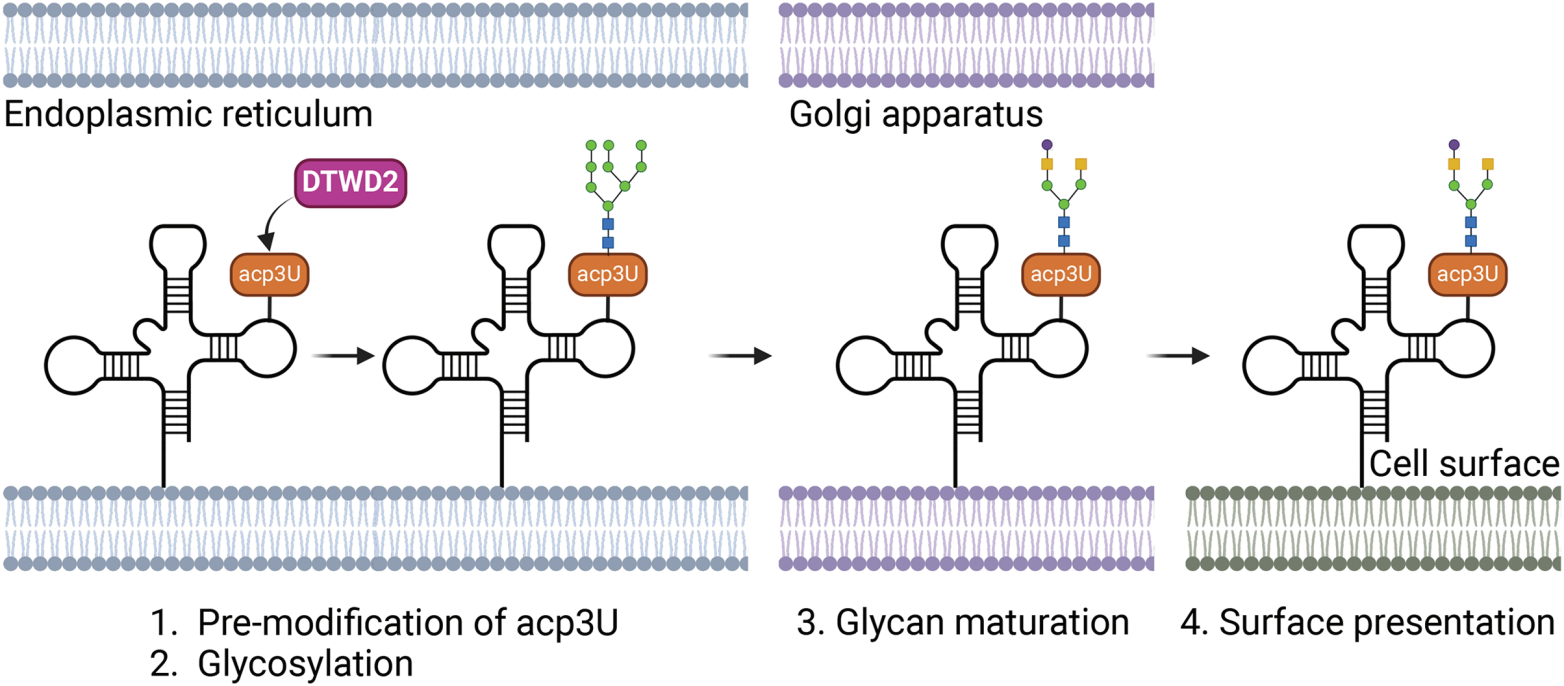
Mechanism of glycoRNA biosynthesis and surface localization. GlycoRNAs undergo a biosynthetic pathway that begins with the modification of specific RNA molecules by the addition of acp3U (3-(3-amino-3-carboxypropyl)uridine), facilitated by the enzyme DTWD2. Following this modification, N-linked glycans are attached to the acp3U site, creating glycoRNAs. These glycoRNAs are subsequently transported through cellular compartments and ultimately displayed on the cell surface.

Using a novel chemical approach called rPAL (RNA-optimized periodate oxidation and aldehyde ligation) combined with sequential window acquisition of all theoretical mass spectra (SWATH-MS), researchers identified 3-(3-amino-3-carboxypropyl)uridine (acp3U) as a critical RNA modification site for N-glycan linkage. acp3U-modified RNAs, primarily tRNAs, are displayed on cell surfaces, where they interact with cellular components and immune molecules. Additionally, enzymes such as DTW domain-containing 2 (DTWD2) are essential for acp3U formation, and their absence significantly alters glycoRNA biosynthesis, reducing glycoRNA display (Fig. 1) [8].

**Figure 2:**
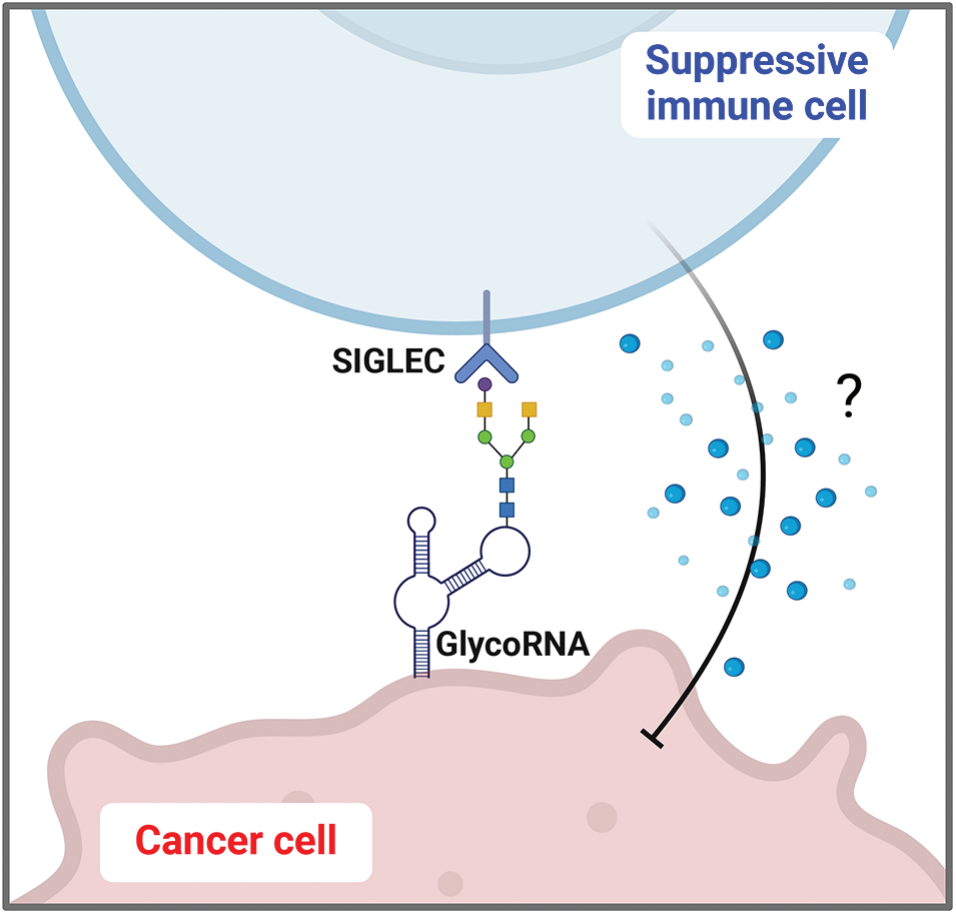
GlycoRNA interaction with SIGLECs (sialic acid-binding immunoglobulin-like lectins) on suppressive immune cells. GlycoRNA displayed on the surface of cancer cells interacts with SIGLEC receptors on suppressive immune cells. By binding to SIGLECs, glycoRNA may trigger inhibitory signaling pathways that contribute to immune suppression within the tumor microenvironment. This interaction enables cancer cells to evade immune detection and promotes tumor growth by dampening the immune response.

The localization of glycoRNAs on the cell surface allows them to interact with glycan-binding proteins, such as sialic acid-binding immunoglobulin-like lectins (SIGLECs). These interactions may have significant implications for immune recognition and response. For instance, the binding of glycoRNAs to SIGLECs on immune cells can transmit inhibitory signals, potentially contributing to immune evasion mechanisms in cancer cells (Fig. 2) [9,10]. Furthermore, glycoRNAs have been shown to bind to anti-double-stranded RNA antibodies, suggesting a role in autoimmune responses. This dual involvement in immune modulation opens new possibilities for the role of glycoRNAs in diseases characterized by immune dysregulation [1]. Notably, recent evidence indicates that cell surface glycoRNAs are essential for neutrophil recruitment and function, underscoring their broad immunological significance beyond cancer and autoimmunity [11]. An emerging feature of glycoRNA biology involves the complementation of these surface interactions by RNA-binding proteins (RBPs) on the cell surface, which form clusters with glycoRNAs and contribute to plasma membrane organization. Cell surface RBPs (csRBPs) have been observed across various cell types, creating specific domains on the cell exterior. The clustering of csRBPs with glycoRNAs may further enhance their interactions with immunomodulatory receptors, facilitating the spatial organization necessary for precise immune recognition and responses [12]. This arrangement of glycoRNA-csRBP complexes on the cell surface could be critical not only for immune modulation but also for broader structural functions on the cell membrane. Understanding the mechanisms governing these interactions and their disease implications, including cancer, remains an active area of research.

## Implications in Cancer Biology

In cancer, glycoRNAs may contribute to various aspects of tumor development and progression. Recent studies using sialic acid aptamers and RNA *in situ* hybridization-mediated proximity ligation assays (ARPLA) have revealed that surface glycoRNA levels are inversely associated with tumor malignancy and metastasis in cancer cell lines [13]. Specifically, non-tumorigenic breast cells exhibited higher glycoRNA abundance compared to that in malignant and metastatic breast cancer cells, which showed progressively lower glycoRNA signals. This suggests that decreased glycoRNA expression may be linked to increased tumor aggressiveness.

Additionally, RNA glycosylation may influence cancer cell progression. Glycosylation patterns play critical roles in cancer progression by affecting cell adhesion, migration, and interactions with the tumor microenvironment [2]. For instance, in epithelial cancers, aberrant glycosylation of the cell adhesion molecule E-cadherin—an essential component for maintaining cell-cell adhesion and tissue architecture—disrupts its normal function and facilitates tumor progression. In gastric and breast cancers, overactivity of N-acetylglucosaminyltransferase V leads to the addition of extra N-acetylglucosamine branches to the N-glycans attached to E-cadherin [14,15]. Abnormal glycosylation alters the conformation and function of E-cadherin, weakening its adhesive properties and enabling cancer cells to detach more easily from primary tumors. Similarly, glycoRNAs may interact with stromal cells and contribute to the remodeling of the tumor microenvironment, promoting tumor growth and resistance to therapy. Changes in glycoRNA expression can affect glycan-mediated signaling pathways that influence angiogenesis, immune cell recruitment, and stromal cell activation.

**Figure 3:**
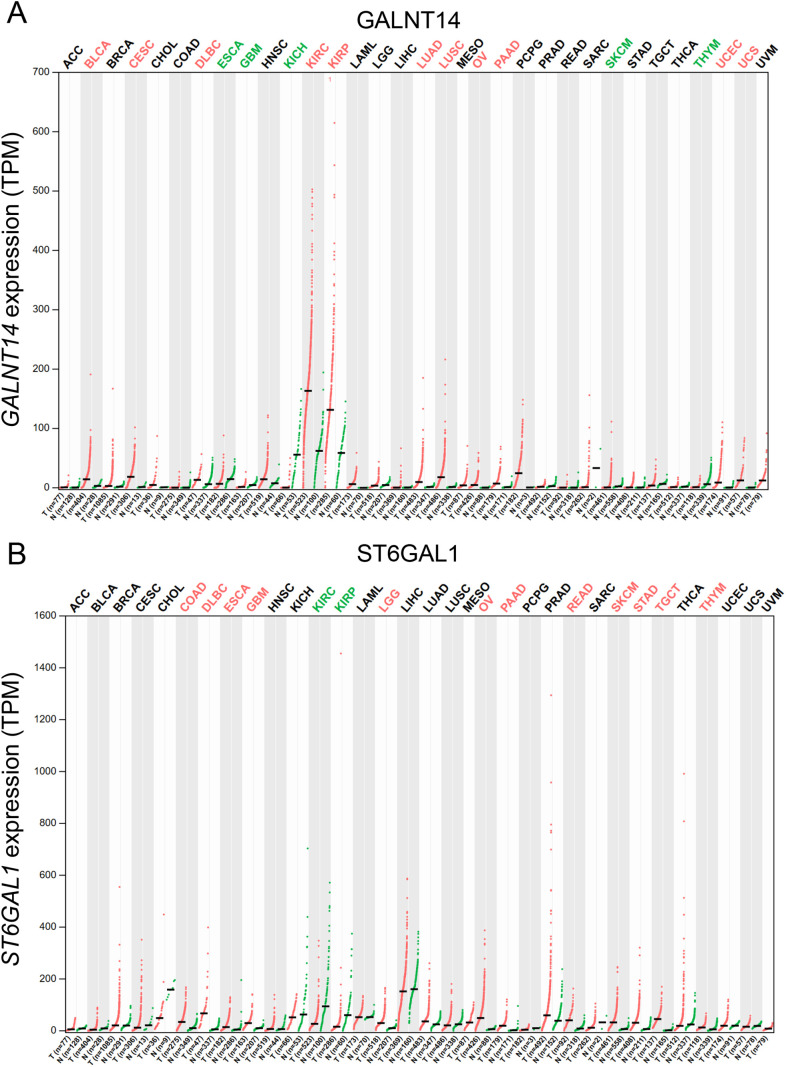
Expression levels of glycoRNA-related genes across various cancer types. (A) and (B) display gene expression data for GALNT14 and ST6GAL1, respectively, analyzed using TCGA (The Cancer Genome Atlas) pan-cancer datasets. Cancer types in red indicate gene overexpression, while those in green represent gene underexpression. ACC, adrenocortical carcinoma; BLCA, bladder urothelial carcinoma; BRCA, breast invasive carcinoma; CESC, cervical squamous cell carcinoma and endocervical adenocarcinoma; CHOL, cholangiocarcinoma; COAD, colon adenocarcinoma; DLBC, lymphoid neoplasm diffuse large B-cell lymphoma; ESCA, esophageal carcinoma; GBM, glioblastoma multiforme; HNSC, head and neck squamous cell carcinoma; KICH, kidney chromophobe; KIRC, kidney renal clear cell carcinoma; KIRP, kidney renal papillary cell carcinoma; LAML, acute myeloid leukemia; LGG, brain lower grade glioma; LIHC, liver hepatocellular carcinoma; LUAD, lung adenocarcinoma; LUSC, lung squamous cell carcinoma; MESO, mesothelioma; OV, ovarian serous cystadenocarcinoma; PAAD, pancreatic adenocarcinoma; PCPG, pheochromocytoma and paraganglioma; PRAD, prostate adenocarcinoma; READ, rectum adenocarcinoma; SARC, sarcoma; SKCM, skin cutaneous melanoma; STAD, stomach adenocarcinoma; TGCT, testicular germ cell tumors; THCA, thyroid carcinoma; THYM, thymoma; UCEC, uterine corpus endometrial carcinoma; UCS, uterine carcinosarcoma; UVM, uveal melanoma.

Altered expression of glycoRNA-related enzymes in cancer cells may also contribute to multiple facets of tumor progression. Enzymes involved in glycosylation, such as GALNTs and sialyltransferases, are often aberrantly regulated in tumors and are associated with poor prognosis. For example, GALNT14, which influences O-glycosylation patterns, and ST6GAL1, an enzyme that adds sialic acid residues for N-glycosylation, are dysregulated in various cancers (Fig. 3A,B) [16,17]. Due to their abnormal expression profiles in malignancies, GALNT14 and ST6GAL1 are promising candidates for further investigation. Further studies are needed to explore their impact on glycoRNA regulation and how these changes may contribute to tumorigenesis. By affecting glycoRNA synthesis or altering glycan composition, cancer cells may gain new mechanisms to regulate gene expression post-transcriptionally, thereby influencing various pathways involved in cancer progression.

Understanding these interactions could provide insight into how tumors manipulate their surroundings and help identify new targets for disrupting these processes. The development of imaging methods, such as ARPLA, not only enables the visualization of glycoRNA spatial distributions but also opens avenues for exploring their functional roles in cancer biology.

## Therapeutic Horizons

The elucidation of glycoRNA function presents several promising avenues for therapeutic development. Targeting enzymes involved in glycoRNA biosynthesis, such as GALNTs and sialyltransferases, could manipulate the production of glycoRNAs, potentially restoring immune recognition and inhibiting tumor growth. Similarly, the inhibition of glycoRNA synthesis may disrupt mechanisms by which cancer cells evade the immune system.

Another potential strategy involves blocking the interactions between glycoRNAs and their binding partners. The development of molecules that interfere with these interactions could enhance immune responses against cancer cells. Monoclonal antibodies or small-molecule inhibitors may be designed to prevent glycoRNAs from interacting with immune inhibitory receptors.

Therefore, glycoRNAs may serve as novel biomarkers for cancer diagnosis and prognosis. Their unique presence in cancer cells and involvement in tumor-specific pathways make them attractive targets for diagnostic assays and imaging techniques. The detection of glycoRNAs could aid in early cancer detection, monitoring disease progression, and evaluating responses to therapy. Combining glycoRNA-targeted therapies with existing immunotherapies, such as immune checkpoint inhibitors, may produce synergistic effects and improve patient outcomes. Integrating these strategies could lead to the development of more effective treatments that address the complexities of tumor biology and immune interactions.

However, ensuring therapeutic specificity remains paramount. Off-target effects could arise if glycoRNA-directed treatments affect healthy cells or disrupt normal glycosylation pathways. To mitigate this, advanced delivery systems, such as nanoparticle-based vehicles and ligand-specific targeting strategies (e.g., antibodies or aptamers recognizing tumor-associated markers), could focus therapeutic agents on malignant cells while sparing normal tissues. Such refinements in targeting and delivery will be critical not only for cancer therapies but also for the application of glycoRNA-based strategies in other clinical contexts.

In addition to cancer, the exploration of glycoRNAs in other disease contexts, such as autoimmune disorders and metabolic diseases, presents additional opportunities. The analysis of glycoRNA patterns in patient-derived samples, including exosomes released during immune activation, could reveal disease-specific signatures and serve as diagnostic or prognostic biomarkers. These insights could not only facilitate earlier and more accurate diagnosis but also enable precise, personalized treatment strategies tailored to an individual’s glycoRNA profile. Over time, this approach could extend the therapeutic potential of glycoRNAs from oncology to a broader range of pathologies, ultimately supporting a more integrative and patient-centric healthcare model.

## Challenges and Future Directions

Although the therapeutic potential of targeting glycoRNAs is significant, several challenges must be addressed. First, our understanding of glycoRNA biology remains in its early stages, underscoring the need for further research to elucidate the mechanisms by which glycoRNAs are synthesized, transported to the cell surface, and interact with other molecules. In particular, employing advanced spatial transcriptomics and high-resolution imaging techniques, such as single-molecule fluorescence *in situ* hybridization (smFISH) and multiplexed error-robust fluorescence *in situ* hybridization (MERFISH), could provide critical insights into the cellular and subcellular localization of glycoRNAs, revealing how they shape tumor heterogeneity and immune interactions [18,19]. Only through such foundational studies can safe and targeted therapeutic interventions be effectively designed. Another key challenge is ensuring therapeutic specificity. Given that glycosylation is a fundamental cellular process, its indiscriminate inhibition can lead to unintended side effects affecting normal cellular functions. Therefore, therapies targeting glycoRNAs must be carefully designed to minimize off-target effects. This might involve the use of RNA-binding protein-specific inhibitors or glycoRNA-targeted aptamers, as well as employing sophisticated delivery methods that harness nanotechnology and other targeted vehicles to direct treatments specifically toward malignant cells. Developing targeted delivery systems for cancer cells is crucial for achieving this specificity while protecting normal tissues.

Advances in delivery technologies, such as nanotechnology and targeted delivery vehicles, are essential for enhancing the precision and effectiveness of glycoRNA-targeted treatments. The integration of these delivery platforms with emerging diagnostic tools and glycoRNA-based biomarkers may further refine patient stratification and improve therapeutic responsiveness. These approaches offer promising solutions for delivering therapeutics directly to tumor cells. Finally, rigorous preclinical studies and subsequent clinical trials are required to validate the efficacy and safety of glycoRNA-based therapies. Progress in this field will undoubtedly benefit from interdisciplinary collaboration across molecular biology, oncology, immunology, and glycobiology, as well as the application of spatially resolved transcriptomic techniques, which together can accelerate the translation of glycoRNA research into impactful treatments.

Considering the potential significance of glycoRNAs in various biological processes, future studies should explore their developmental trajectories in various organs and tissues. Utilizing large-scale transcriptome datasets, such as those from the Genotype-Tissue Expression project (GTEx) Consortium, can reveal how glycoRNA expression patterns change over time and in different physiological contexts [20]. Such data may reveal organ-specific roles and critical developmental windows where glycoRNAs influence disease progression, ultimately guiding more precise and temporally targeted therapeutic interventions.

## Conclusion

The emergence of glycoRNAs as novel factors in cellular biology signifies a transformative shift, with profound implications for cancer therapy and immune modulation. By revealing new mechanisms of cellular communication and offering potential therapeutic targets, glycoRNAs hold promise for developing treatments that can disrupt cancer progression and improve patient outcomes. As glycoRNA research progresses, incorporating these findings into cancer therapies and immune-targeted strategies may revolutionize treatment paradigms, addressing the complexities of tumor biology and immune interactions. The biomedical field is on the cusp of significant advancements, with glycoRNAs emerging as a promising avenue for more effective and targeted treatments for challenging diseases.

## Data Availability

Data sharing not applicable to this article as no datasets were generated or analyzed during the current study.

## Abbreviations

acp3U: 3-(3-Amino-3-Carboxypropyl) Uridine
ARPLA: Sialic Acid Aptamer and RNA *In Situ* Hybridization-Mediated Proximity Ligation Assay
csRBPs: Cell Surface RNA-Binding Proteins
DTWD2: DTW Domain Containing 2
ER: Endoplasmic Reticulum
GALNTs: N-Acetylgalactosaminyltransferases
GTEx: The Genotype-Tissue Expression Consortium
MERFISH: Multiplexed Error-Robust Fluorescence *In Situ* Hybridization
RBPs: RNA-Binding Proteins
rPAL: RNA-Optimized Periodate Oxidation and Aldehyde Ligation
SIGLECs: Sialic Acid-Binding Immunoglobulin-Like Lectins
smFISH: Single-molecule fluorescence *in situ* hybridization
ST6GAL1: β-Galactoside α-2,6-Sialyltransferase 1
SWATH-MS: Sequential Window Acquisition of all Theoretical Mass Spectra
TCGA: The Cancer Genome Atlas

## References

[1] Flynn RA, Pedram K, Malaker SA, Batista PJ, Smith BAH, Johnson AG, et al. Small RNAs are modified with N-glycans and displayed on the surface of living cells. Cell. 2021;184(12):3109–24.E22. 34004145 10.1016/j.cell.2021.04.023 PMC9097497

[2] Pinho SS, Reis CA. Glycosylation in cancer: mechanisms and clinical implications. Nat Rev Cancer. 2015;15(9):540–55. doi:10.1038/nrc3982; 26289314

[3] Ren X, Lin S, Guan F, Kang H. Glycosylation targeting: a paradigm shift in cancer immunotherapy. Int J Biol Sci. 2024;20(7):2607–21. doi:10.7150/ijbs.93806; 38725856 PMC11077373

[4] Mohorko E, Glockshuber R, Aebi M. Oligosaccharyltransferase: the central enzyme of N-linked protein glycosylation. J Inherit Metab Dis. 2011;34(4):869–78. doi:10.1007/s10545-011-9337-1; 21614585

[5] O’Brien K, Breyne K, Ughetto S, Laurent LC, Breakefield XO. RNA delivery by extracellular vesicles in mammalian cells and its applications. Nat Rev Mol Cell Biol. 2020;21(10):585–606. doi:10.1038/s41580-020-0251-y; 32457507 PMC7249041

[6] Duca M, Malagolini N, Dall’Olio F. The mutual relationship between glycosylation and non-coding RNAs in cancer and other physio-pathological conditions. Int J Mol Sci. 2022;23(24):15804. doi:10.3390/ijms232415804; 36555445 PMC9781064

[7] Sachse M, Stellos K. Unraveling the RNA code: a uridine RNA modification drives glycoRNA biogenesis. Signal Transduct Target Ther. 2024;9(1):334. doi:10.1038/s41392-024-02056-z; 39604375 PMC11603334

[8] Xie Y, Chai P, Till NA, Hemberger H, Lebedenko CG, Porat J, et al. The modified RNA base acp^3^U is an attachment site for N-glycans in glycoRNA. Cell. 2024;187(19):5228–37.E12. 39173631 10.1016/j.cell.2024.07.044 PMC11571744

[9] Clyde D. Sugar-coated RNAs. Nat Rev Genet. 2021;22(8):480. doi:10.1038/s41576-021-00388-y; 34168329

[10] Fu M, Qian Y, Huang E, Schwarz Z, Tai H, Tillock K, et al. GlycoRNA-L and glycoRNA-S mediate human monocyte adhesion via binding to Siglec-5. 2024. doi:10.1101/2024.08.27.609838. PMC12281301 40609958

[11] Zhang N, Tang W, Torres L, Wang X, Ajaj Y, Zhu L, et al. Cell surface RNAs control neutrophil recruitment. Cell. 2024;187(4):846–60 e17. 38262409 10.1016/j.cell.2023.12.033 PMC10922858

[12] Perr J, Langen A, Almahayni K, Nestola G, Chai P, Lebedenko CG, et al. RNA binding proteins and glycoRNAs form domains on the cell surface for cell penetrating peptide entry. 2023. doi:10.1101/2023.09.04.556039. PMC11972160 40020667

[13] Ma Y, Guo W, Mou Q, Shao X, Lyu M, Garcia V, et al. Spatial imaging of glycoRNA in single cells with ARPLA. Nat Biotechnol. 2024;42(4):608–16. doi:10.1038/s41587-023-01801-z; 37217750 PMC10663388

[14] Pinho SS, Figueiredo J, Cabral J, Carvalho S, Dourado J, Magalhaes A, et al. E-cadherin and adherens-junctions stability in gastric carcinoma: functional implications of glycosyltransferases involving N-glycan branching biosynthesis, N-acetylglucosaminyltransferases III and V. Biochim Biophys Acta. 2013;1830(3):2690–700. doi:10.1016/j.bbagen.2012.10.021; 23671930

[15] Pinho SS, Seruca R, Gartner F, Yamaguchi Y, Gu J, Taniguchi N, et al. Modulation of E-cadherin function and dysfunction by N-glycosylation. Cell Mol Life Sci. 2011;68(6):1011–20. doi:10.1007/s00018-010-0595-0; 21104290 PMC11114786

[16] Lin WR, Yeh CT. GALNT14: an emerging marker capable of predicting therapeutic outcomes in multiple cancers. Int J Mol Sci. 2020;21(4):1491. doi:10.3390/ijms21041491; 32098271 PMC7073045

[17] Garnham R, Scott E, Livermore KE, Munkley J. ST6GAL1: a key player in cancer. Oncol Lett. 2019;18(2):983–9; 31423157 10.3892/ol.2019.10458 PMC6607188

[18] Jin Y, Zuo Y, Li G, Liu W, Pan Y, Fan T, et al. Advances in spatial transcriptomics and its applications in cancer research. Mol Cancer. 2024;23(1):129. doi:10.1186/s12943-024-02040-9; 38902727 PMC11188176

[19] Yu Q, Jiang M, Wu L. Spatial transcriptomics technology in cancer research. Front Oncol. 2022;12:1019111. doi:10.3389/fonc.2022.1019111; 36313703 PMC9606570

[20] Consortium GT. The GTEx Consortium atlas of genetic regulatory effects across human tissues. Science. 2020;369(6509):1318–30. doi:10.1126/science.aaz1776; 32913098 PMC7737656

